# Lay perceptions of predictive testing for diabetes based on DNA test results versus family history assessment: a focus group study

**DOI:** 10.1186/1471-2458-11-535

**Published:** 2011-07-05

**Authors:** Miranda Wijdenes-Pijl, Wybo J Dondorp, Danielle RM Timmermans, Martina C Cornel, Lidewij Henneman

**Affiliations:** 1 Department of Public and Occupational Health, EMGO Institute for Health and Care Research, VU University Medical Center, Amsterdam, The Netherlands; 2 Department of Health, Ethics and Society, Research Institute CAPHRI, Maastricht University, Maastricht, The Netherlands; 3 Department of Clinical Genetics, Section Community Genetics, EMGO Institute for Health and Care Research, VU University Medical Center, Amsterdam, The Netherlands

## Abstract

**Background:**

This study assessed lay perceptions of issues related to predictive genetic testing for multifactorial diseases. These perceived issues may differ from the "classic" issues, e.g. autonomy, discrimination, and psychological harm that are considered important in predictive testing for monogenic disorders. In this study, type 2 diabetes was used as an example, and perceptions with regard to predictive testing based on DNA test results and family history assessment were compared.

**Methods:**

Eight focus group interviews were held with 45 individuals aged 35-70 years with (n = 3) and without (n = 1) a family history of diabetes, mixed groups of these two (n = 2), and diabetes patients (n = 2). All interviews were transcribed and analysed using Atlas-ti.

**Results:**

Most participants believed in the ability of a predictive test to identify people at risk for diabetes and to motivate preventive behaviour. Different reasons underlying motivation were considered when comparing DNA test results and a family history risk assessment. A perceived drawback of DNA testing was that diabetes was considered not severe enough for this type of risk assessment. In addition, diabetes family history assessment was not considered useful by some participants, since there are also other risk factors involved, not everyone has a diabetes family history or knows their family history, and it might have a negative influence on family relations. Respect for autonomy of individuals was emphasized more with regard to DNA testing than family history assessment. Other issues such as psychological harm, discrimination, and privacy were only briefly mentioned for both tests.

**Conclusion:**

The results suggest that most participants believe a predictive genetic test could be used in the prevention of multifactorial disorders, such as diabetes, but indicate points to consider before both these tests are applied. These considerations differ with regard to the method of assessment (DNA test or obtaining family history) and also differ from monogenic disorders.

## Background

As a result of genomics research, a growing number of genetic variants that contribute to the multifactorial aetiology of many common disorders, such as diabetes and coronary heart disease, are being identified. Multifactorial diseases are, however, characterised by complex gene-environment interactions [1]. Testing based on these genetic variants alone (DNA-based test), or in addition to traditional disease risk factors, such as obesity and hypertension, still shows limited predictive value for disease [2,3]. While we await the identification of more genetic variants that do show higher predictive value together, family history might be used as a 'genomics' tool for disease prevention [4]. Numerous studies show that familial risk is an important and independent risk factor for multifactorial diseases [5]. Family history reflects the consequences of a genetic predisposition, a shared environment, and common behaviour. Family history information may be used to determine personal disease risk (family history assessment), raise risk awareness and motivate individuals to adopt preventive behaviour [6]. So far, there is little attention for the use of genetic information (DNA test results or a family history assessment) in prevention programmes for common diseases [7]. Nevertheless, new developments in predictive medicine are expected, and this calls for an understanding of issues related to these tests [8].

Little is known about how people compare and contrast predictive testing based on DNA test results to family history assessment, e.g. in terms of perceived utility or perceived drawbacks, and how they compare genetic tests for multifactorial diseases to monogenic diseases. So far, most predictive genetic tests have been used to detect predispositions for single-gene diseases with a strong genetic influence, such as hereditary forms of cancer. For these monogenic diseases there are many data available on the ethical, legal and social issues of testing for and communication of genetic risk information to individuals and families. Important issues are, for example, the right (not) to know one's genetic status [9], freedom of choice, indicating that free and informed consent has to be guaranteed before a genetic test is carried out [8], potential genetic discrimination by insurance companies and employers [10], and privacy issues concerning who has access to sensitive personal genetic information [11]. Yet, limited research has focused on the possible ethical, legal and social issues related to genetic testing for multifactorial diseases [12]. These issues are expected to be less pronounced, because the aetiology is essentially different. Each gene variation might have an effect on more than one disease or phenotype, the inheritance of an identical pattern of gene variants is low, and there is a high environmental influence on the development of a disease [13]. New issues may, however, arise. For example, Janssens and Khoury [13] have expressed their concern about whether it is ethical to perform DNA tests with low predictive value. Other issues may be relevant for family history assessment. For example, Yoon et al. [4] suggested that labelling a family at risk might induce feelings of blame, or induce anxiety associated with knowledge of affected relatives. Moreover, there is still little evidence about the clinical utility of DNA testing and family history assessment for multifactorial diseases [4,14], i.e. how likely is the test to significantly improve patient outcomes and motivate people to engage in preventative behaviour to reduce their disease risk [15].

In this study we used type 2 diabetes as an example of a multifactorial disease, and focussed on both DNA test results and family history assessment to predict the risk for developing diabetes. Diabetes is an important health problem, which has an increasing prevalence due to physical inactivity and unhealthy diet [16]. It has been shown that modest changes in lifestyle can delay or even prevent the onset of diabetes in high risk populations [17]. In addition to behavioural factors, genetic factors also influence the development of type 2 diabetes [18], but with limited predictive value [19]. In contrast, a familial risk of diabetes reflects a 2 to 6-fold increase in the odds of developing diabetes, depending on the number and closeness of affected relatives [20,21].

In order to be able to develop effective prevention programmes for multifactorial diseases, such as diabetes, based on genetic risk information, it is important to explore the opinions and expectations of potential users. Therefore the aim of this study was to asses lay perceptions of issues related to predictive genetic testing (DNA test results or family history assessment) in diabetes prevention.

## Methods

To gain insight into lay perceptions of predictive testing for diabetes, focus group interviews were held. Focus groups aim to promote a variety of opinions and self-disclosures, i.e. group members influence each other by responding to comments made by others [22]. The Medical Ethics Committee of the VU University Medical Center approved the study protocol.

### Participants and procedure

Eight focus group interviews were held with 45 individuals with (n = 3), and without (n = 1) a family history of diabetes, mixed groups of these two (n = 2) and diabetes patients (n = 2). Participants were recruited by means of an advertisement in a regional newspaper, inviting people between 35 and 70 years of age to participate in a group discussion about the prevention of diabetes. It was indicated that they would receive an incentive of €25 (gift card) for participation. No further information was provided. The responders were asked via the telephone whether they had diabetes, and subsequently whether they had a first-degree relative with diabetes. Additionally, one group with a family history of diabetes was recruited among participants in an earlier diabetes screening study in 1999 [23]. In that study, participants had been informed by letter that they did not have diabetes, but no further information about diabetes prevention was given. The diabetes patients were primarily recruited by means of an advertisement in a magazine distributed by the regional Diabetes Patient Organisation. All participants gave written informed consent before participation.

All the focus groups were facilitated by the same moderator (LH), and an assistant (MWP) made notes during each session. The focus groups lasted for approximately 90 minutes, and were held at the University Medical Center and a community facility between June and October 2008. Since the questions addressed in the focus groups required some knowledge about the concepts that were referred to, these were briefly explained at the start of the session using a Powerpoint presentation. The presentation included information about diabetes, such as causes and consequences, and information about the difference between diabetes risk assessment and a blood glucose test to indicate diabetes. Table 1 shows the information that was given about the DNA test and the family history assessment. It was emphasised that the aim of both tests is to inform people about an increased risk for developing diabetes and to provide them with preventive options to reduce the risk, i.e. a healthy diet and physical activity. No specific risk percentages were given.

**Table 1:** Information given to participants during the focus group sessions concerning both genetic risk assessments

DNA test	Family history assessment
- Risk is increased by genetic predisposition	- Includes genetic predisposition, common behaviour, and shared environment
- Assessed by taking a sample of blood or saliva	- Risk increases with number and closeness of affected family members
- Test result does not depend on the occurrence of diabetes within the family	- Assessed by asking about the number and relatedness of affected family members

A semi-structured interview guide was used to assess perceptions. The participants were asked to indicate the possible advantages, disadvantages, and barriers related to both tests in diabetes prevention. Subsequently, the effect on individuals and their families (stigmatisation, discrimination, worry), and privacy issues when using genetic risk information in diabetes prevention were introduced if the participants did not bring these subjects up (see Additional file 1). In addition, two hypothetical vignettes were used to stimulate discussion. The vignettes described two males aged 55 years with a high risk of developing diabetes as a result of: a) two first-degree family members with diabetes (family history assessment), and b) a positive test result on a DNA test for diabetes (DNA test). The participants were asked to indicate which of these two men would be most or least motivated to adopt healthy behaviour, and why. Characteristics of the participants in the focus groups were obtained by means of a brief self-completed questionnaire (see Table 2).

**Table 2:** Characteristics of the participants (n = 45) in the focus groups (n = 8)

Group code	Average age in years (SD)	Total	Gender	Level of education*			Family history of diabetes**		
			Female	Low	Intermediate	High	1^st^ degree	2^nd^ degree	None
		n	n	n	n	n	n	n	n
1NoFam, general population	52 (7)	6	5	0	3	3	0	0	6
1Fam, family history	55 (11)	5	3	0	2	3	4	1	0
2Fam, family history	46 (23)	3	3	0	2	1	2	1	0
3Fam, family history	66 (3)	7	5	5	1	1	6	0	1***
1Mix, mixed group	63 (6)	5	5	1	3	1	1	1	3
2Mix, mixed group	52 (11)	8	6	1	2	5	1	1	6
1Pt, patients	57 (8)	7	5	0	5	2	7	0	0
2Pt, patients	55 (9)	4	2	0	2	2	1	1	2
Total	56 (11)	45	34	7	20	18	22	5	18

* Low level of education refers to people who completed elementary school, lower secondary education or lower vocational education; Intermediate level of education refers to higher secondary education or intermediate vocational education; High level of education refers to university or higher vocational education

** 1^st ^degree refers to having at least one or more first degree family member(s) with diabetes; 2^nd ^degree refers to having at least one or more second degree family member(s) with diabetes

*** This person had an extensive family history of people with cardiovascular diseases, but no family members with diabetes.

### Preparation of data and analyses

All focus group interviews were audio-taped and transcribed. Atlas-ti software was used for the analyses. First, codings were annotated to the data to structure and analyse the transcripts. The codings were then clustered to define sub-themes and main themes. On the basis of these themes, further analyses were performed to detect and check correspondence and differences between the themes, and to search for the most important messages transmitted by the participants. In order to ensure uniform coding, two authors (MP and LH) coded each transcript, and then discussed the codings until agreement was reached [24]. In the results, quotations are used to illustrate the meanings that participants attached to a theme. Quotations were translated from Dutch and checked by a Dutch to English translator. Characteristics of participants are given in brackets, indicating the type of group (group number (1-3), general population of people with no family history, with a family history, mixed group or diabetes patients (NoFam, Fam, Mix, Pt), [family members with diabetes], gender (male, female), and age in years).

## Results

The most important themes that emerged concerning predictive genetic testing for diabetes were: 1) identification of people at risk, 2) positive and negative health outcomes, 3) family issues, 4) informational privacy, and 5) autonomy. These themes and corresponding sub-themes are shown in Table 3. Perceived differences between testing based on DNA test results and family history assessment will be illustrated.

**Table 3:** Overview of themes raised by participants comparing DNA test results with family history assessment

*Themes*	*DNA test*	*Family history assessment*	*Both tests*
**Identification of people at risk**			
	Diabetes is not severe enough	Can be assessed quickly	Can identify people at risk
		Diabetes family history is unknown	Only for high risk individuals
		No use for people with no family history	
		There are other risk factors for diabetes	
**Positive and negative health outcomes**			
Motivation to engage in healthy behaviour	The test is a deliberate decision	There is an example of diabetes patients within the family	Genetic risk cannot be influenced
	Risk is certain	People with a family history are already aware of the risk	False reassurance
Psychological impact		Worry for and about children	Little or no psychological harm
			Worry about diabetes risk
**Family issues**			
Influence on family relationships		Opens family discussion about diabetes and provides support	
		Some do not want to be informed, disturbs family relations	
		Someone in the family will be blamed	
Informing family members			Obligation to disclose risk information to family members
			Allows to raise children more consciously
**Autonomy**			
	Not performed unasked for	Can be offered to everyone	Risk tests should be voluntary
	No tests on embryos or children		
	Informative before having children		
**Informational privacy**			
Discrimination			Discrimination by insurance company or employer
Sensitive data	Ownership of data	Private information	
	No trust in relatively new test		

### Identification of people at risk

Participants supported both the use of DNA tests and family history assessment in order to identify people who are at risk for diabetes:

If diabetes runs in your family, you don't assume that you can get it [diabetes] too. If you have a predisposition for diabetes, because family members are affected, then I think it is a good idea to promote such a test [family history assessment], because not everyone will perceive the risk. (2Mix [nephew], male, 43 years)

You know it instantly [whether you're at risk], by taking some blood. Do I have a predisposition, yes or no? Brief and effective; it's [DNA test] a good test. (1NoFam, female, 53 years)

However, some believed diabetes was not severe enough for DNA testing:

Genetic testing is [more than for diabetes] for serious diseases like cystic fibrosis, cancer, kidney diseases. Having a family member with one of these diseases can be a reason to have a genetic test. (1Mix [grandmother], female, 69 years)

Participants indicated the advantage that taking a family history can be done quickly. Others questioned this assessment, because information about the presence of diabetes within the family is not always known, it is of no use for people without a family history, and there are other risk factors for diabetes apart from family history:

Having a family member with diabetes implies an increased risk, but it's limited, information on unhealthy living and physical activity should also be included. (2Mix, female, 52 years)

Some participants emphasised that there should be a reason for risk assessment to be most effective, i.e. that people should be at increased risk before having their risk assessed.

I don't think that people will think it concerns them. People will only respond to risk information when they have physical complaints and only then they will think "Now I have to be careful.". (2Fam [mother], female, 65 years)

### Positive and negative health outcomes

#### Motivation to adopt healthy behaviour

A positive outcome of predictive testing for diabetes that was mentioned was that it may motivate to engage in healthy behaviour. Although many participants experienced no difference between DNA test results and family history assessment in the effect on people's motivation to adopt healthy behaviour, some participants believed that people would be more motivated to adopt risk-reducing behaviour after having had a DNA test. The underlying reason they gave was that to have a DNA test is a deliberate decision, since the first step towards risk reduction has already been taken by having such a test. In addition, this risk was seen as most certain:

The DNA test gives the hardest 'push' to live healthier. It will frighten me more than a test based on a family history, because it is the strongest evidence. (1Pt [father, 2 brothers], male, 51 years)

Others believed that familial risk information will motivate people more than a DNA test to adopt healthy behaviour, since these people see examples (of the consequences) of diabetes within their family. The contrary of enhancing motivation, however, was also mentioned. Some believed that a family history assessment will not increase the motivation of people with a familial risk, since these people will already be aware of the risk. It was also believed that for some people genetic risk information, based either on DNA or family history, could reduce motivation. Here an example of the adverse impact of family history assessment is given:

If people are informed that they have a risk, because their father had diabetes, and their grandfather, grandmother, and aunt too; it's discouraging. They will accept the risk and think they can't prevent diabetes, since it's heritable. (2Pt, male, 56 years)

Furthermore, some participants mentioned that people could be falsely reassured if they are told that they have no genetic risk.

There is a chance that if people hear that they have no predisposition [for diabetes], that they will think "I can eat what I want, being fat is no problem, and being physically active, I prefer sitting behind the computer all day.". (1NoFam, female, 57 years)

#### Psychological impact

Although some believed that knowing the disease risk could induce worry, most participants believed that diabetes risk assessment, even when genetic risk assessment is used, would cause very little or no psychological harm. Moreover the effect on raising risk awareness was emphasised:

Familial risk information will not necessarily lead to worry [about disease risk], but it can raise awareness about the risk. (1Fam [mother, brother], female, 64 years)

Others, however, thought that people might be worried for and about their children, when family history will be assessed:

A [familial] risk can be threatening. I've got a 14 year-old daughter. If she would be tested [family history assessment] and hears that she has an increased risk for diabetes, too, that would be a kind of "Damocles' sword" hanging above her head. (1Pt [mother], female, 50 years)

### Family issues

#### Impact on family relationships

Participants discussed the possible impact of familial risk information on family relations. On the one hand, some believed that it opens up discussions, and can be used to support each other to adopt healthy behaviour. For example, this woman (diabetes is prevalent in her husband's family) said:

I think that when you're aware of diabetes running in your family, it can help to talk about it with each other. I notice that the relationships in our family are not distorted. We talk about it [diabetes in the family] with each other. Not that we get anxious about it, but more to be supportive for other family members. It's no longer a taboo. (1NoFam, female, 47 years)

On the other hand, some thought that it could also disturb family relationships if family members do not want to be informed about their diabetes risk:

My relatives will get anxious [if our family history is assessed]. [...] There are some who would rather not know that. So, I think that it can cause anxiety for some people. (1Mix, female, 57 years)

One participant mentioned that, if family members are identified to be at risk for diabetes, the patient with diabetes in the family might be blamed for putting the family at risk.

#### Informing family members

Some diabetes patients felt the obligation to disclose risk information to other members of the family:

I warned my brother and he visited his general practitioner. He had it, in a less severe way, nevertheless. So, I think it's good to warn family members. (1Pt, [father, mother, brother, uncle, aunt], male, 63 years)

Another issue was that participants, diabetes patients in particular, did not only see the benefit of genetic risk information for themselves, but indicated that knowing their risk will also allow people to raise their children more consciously:

[DNA test results] can be used preventatively, if I know that my little son might be at risk I can be careful about his diet. (2Pt [father, grandmother], female, 46 years)

### Autonomy

Issues that would affect autonomy were emphasised more when the DNA test was discussed. While most participants believed that DNA tests should never be performed (or even offered) unless asked for, no such condition was suggested with regard to a family history assessment. Moreover, only one participant mentioned that having a family history assessment should be voluntary. For this test it was even suggested that it should be actively offered to the entire population:

Why are we never approached for such a test [family history assessment]? Now, people themselves must request it, but they don't go to the doctor for such a thing. [...] So, I think that the government should provide the test.

Moreover, one woman also mentioned that DNA tests should not be performed on embryos or children, possibly referring to the fact that they are not able to properly consent:

They might test [DNA test] young children, and they may even test embryos [...] there is a high risk that they will go further, and one thing may lead to another. (1Fam [mother], female, 58 years)

Another person mentioned that a DNA test could be informative before having children, suggesting that it can be used before conception to give an indication of a possible genetic predisposition for diabetes in the offspring, and thus as a means to reproductive autonomy:

For example, for young people who would like to have children, and diabetes is very common in their family. I think it's a good thing that they can have a genetic test. (2Fam [mother], female, 65 years)

### Informational privacy

#### Discrimination

Only one participant indicated that a consequence of a genetic test might be discrimination on the basis of the test results:

How will the [family history] information be used? Now it's voluntary to provide the information, but these tests may be obligatory in the future. Then you have the risk that people will be unable to get insurance; life insurance and that sort of thing. (3Fam, female, 61 years)

This woman therefore argued that family information should be protected from third parties. Subsequently this belief was picked up and supported by other participants.

#### Sensitive data

Two participants worried about the safety of the data-storage of DNA test results (ownership of data):

I can imagine that your whole genetic profile will then be public...I think it should be stored somewhere safe. When it's stored with the general practitioner, there is at least some privacy. (2Mix, female, 49 years)

In addition, some did not trust DNA test results, because it is a relatively new test:

If they want to prove that someone is at risk, they can manipulate the results. I don't know how, since it's quite new, quite precarious. (1Mix [grandmother], female, 69 years)

With regard to a family history assessment, a diabetes patient indicated that discussing the presence of diabetes in the family can be considered as private information:

You can get a problem with privacy. When you say my brother has diabetes and my sister has diabetes, and she's too fat, then you say things about your family that are personal, and some people might have a problem with that. (1Pt, male, 51 years)

## Discussion

This study provides an overview of perceived issues that may arise among the public when predictive tests for diabetes, based either on DNA test results or family history assessment, are introduced. Although it was believed that both tests could be used to identify people at risk for diabetes, also drawbacks were mentioned. With regard to the motivational impact, participants perceived differences in the underlying mechanism between a DNA test and family history assessment and both arguments for a positive and a negative impact were mentioned. Respect for autonomy of individuals was emphasized more with regard to DNA testing than family history assessment. Psychological harm, and discrimination, privacy were only mentioned by some participants.

Participants had high expectations about the predictive value of DNA test results. However, until now genetic variants have a marginal improvement in predictive ability above traditional risk factors for diabetes [2,19]. Also, some participants had an unrealistic perception of the possible uses of DNA test results, e.g. they believed that the test result can be manipulated, or believed that information about a genetic risk for diabetes could be used to make a reproductive choice. Earlier studies have shown that many people know little about genetic technology, and have unclear notions about the benefit of genetic testing for multifactorial diseases, but are interested in having these tests [25]. For example, it has been shown that non-diabetic patients were more likely to request a genetic test to assess future diabetes risk than physicians were to recommend it, and also had higher beliefs that it will motivate people to adopt healthy behaviour [26].

Although the participants believed that both assessments could be used to identify high risk individuals, some thought that diabetes was not severe enough for a DNA test. People in general perceive diabetes as less threatening than other common diseases such as cancer or heart disease [27]. Other participants in this study questioned the accuracy and reliability of family history reports, since the presence of diabetes in the family is not always known. An under-reporting of the family history of common chronic diseases for parents and siblings has, indeed, been found in several studies [28].

Participants gave several underlying reasons for the motivational impact of either a DNA test or a family history assessment, and these differed between both tests. Some believed that people would get more motivated after receiving DNA test results, since a first step has already been made by taking such a test and the test result is considered more certain as compared with a family history test. The perception that a diabetes risk based on DNA test results is more concretely defined and based on evidence has previously been identified among people at risk for getting diabetes [29]. With regard to family history assessment, participants in this study mentioned that having examples of diabetes patients within the family will motivate people to engage in preventive behaviour. Research has shown that the perceived difference in impact of genetically-based risk information or family history-based risk information on motivation (or intention) to engage in recommended health behaviour is inconsistent [30,31]. Only few studies evaluated the impact of genetic risk information on motivation (or intention) to adopt healthy behaviour and report conflicting effects on actual behaviour change [13,32]. Some participants in this study believed that genetic risk information could lead to fatalism, i.e. people being less motivated to change their behaviour. However, there is no evidence for such an effect [33].

In the present study, no prominent distinctions were found between the opinions of people with a family history of diabetes, people without a family history of diabetes, and diabetes patients. It seemed, however, that diabetes patients perceived more benefits of genetic risk information for their children compared to the other participants, and also felt obliged to disclose risk information to other members of the family. Also, participants mentioned that familial risk information can support family members to jointly adopt preventative behaviour. It has indeed been shown that healthy eating habits may be easier to achieve if the entire family is involved in promoting healthy living [34].

Participants placed more emphasis on autonomy with regard to the unrequested offer of a DNA-test, as they do not want to be offered such a test unless asked for, whereas this was not brought up as a condition with regard to a family history assessment. Moreover, with regard to a family history assessment, only one person indicated that it will be important to be able to accept or reject the test, thus stressing that participation should be voluntary. In general, we conclude that being offered a family history test was far less readily considered as a potential threat to autonomy than the offer of a DNA-test. Therefore, the former type of assessment might be more ready for a public health setting, as has been suggested by others [4,6]. Khoury [35] indicated that with the output of the human genome project there has been a shift from gene-assessment for individuals or families (monogenic disorders) to a public health approach (multifactorial diseases). While genetics has traditionally focused on a non-directive way of communicating information to diagnose and manage rare conditions for which there might not be effective interventions [36]. Type 2 diabetes is very common and people can reduce their risk for the disease by adopting healthy behaviour. This may justify a public health approach, i.e. offering a family history risk assessment and health messages to people who did not ask for it. Indeed, the issues mentioned by the participants in the present study are comparable to non-genetic risk assessment and related to issues that are specifically relevant in public health.

In this explorative study, issues related to monogenic diseases, such as discrimination, privacy, and psychological impact, were mentioned by only few participants; for example, the possibility of discrimination by insurance company or employer based on genetic test results, privacy or induced worry. A reason for this might be that the views of people, as far as they are familiar with genetic risks, may be influenced by discussions about genetic testing for monogenic disorders. It is, however, expected that issues concerning, for example, diabetes risk worry and discrimination, might be less applicable for multifactorial diseases than for monogenic disorders [12], because of the preventive options that are available. In a review on the delivery of genomic medicine for common chronic diseases it was concluded that there are no well-documented cases of health insurers either asking for or using genetic test results for discriminative purposes [25]. However, even with existing legislation the fear of genetic discrimination with regard to testing for adult-onset diseases has not greatly reduced [37].

The findings presented in this paper are not intended to be generalised, rather they give an overview of possible issues related to genetic risk assessment for diabetes as perceived by participants. It can be expected that people who are more interested in health or who are in need of money are more prone to have responded on the study invitation. Besides, more women than men participated in this study, which may have influenced the findings as it has been shown that women, in general, are less favourable towards genetic testing than men [38,39]. A drawback in the discussions may have been that the current low predictive value of DNA test results was not explained to the participants, since this information was considered to be too complex. Moreover, because of the semi-structured interview guide, the issues that were raised by the participants were also more or less the issues that were addressed during the interviews. Monogenic sub-types of diabetes, such as Maturity Onset Diabetes of the young (MODY), were not considered. The predictive value of genetic testing for these rare sub-types is clearly higher.

## Conclusions

The results of this study indicate that individuals believe in the ability of predictive genetic testing to identify people at diabetic risk and enhancing healthy behaviour, but also points to consider before using these tests were identified. With regard to DNA tests these are, e.g. an unrealistic perception of the test results, and a perceived threat for autonomy. It is therefore important to educate people about DNA tests for multifactorial diseases and to inform possible future consumers about the low predictive value, as was also recommended by the European Society of Human Genetics [11]. With regard to the family history assessment, participants indicated drawbacks in identifying people at risk and a possible negative influence on family relations. The results further show that issues that are important in testing for monogenic diseases, such as privacy, discrimination, and psychological harm, are mentioned by some participants with regard to this multifactorial disease. Nevertheless, new issues will most likely become important, which are more related to a public health setting and non-genetic risk information.

## Competing interests

The authors declare that they have no competing interests.

## Authors' contributions

MWP coordinated the study, carried out the focus group and individual interviews and drafted the manuscript. LH facilitated the focus groups. All authors participated in the design of the study, and read and approved the final manuscript.

## Pre-publication history

The pre-publication history for this paper can be accessed here:

http://www.biomedcentral.com/1471-2458/11/535/prepub

## Supplementary Material

Additional file 1 **Interview guide for the focus groups**. Semi-structured interview guide and vignettes that were used during the focus group interviews with the lay participants. Click here for file
