# Supplementary material for: Lay perceptions of predictive testing for diabetes based on DNA test results versus family history assessment: a focus group study
Source: BMC Public Health. 2011 Jul 5;11:535. doi: 10.1186/1471-2458-11-535 (PMC3155914; doi:10.1186/1471-2458-11-535)
Supplement: Additional file 1 — Interview guide for the focus groups. Semi-structured interview guide and vignettes that were used during the focus group interviews with the lay participants. [file 1471-2458-11-535-S1.DOC]

# Additional file 1

# Title: Interview guide for the focus groups

**Description: Semi-structured interview guide and vignettes that were used during the focus group interviews with the lay participants**

1. What do you think of a diabetes risk test that uses a [family history assessment/DNA test] to indicate an increased risk for diabetes?

2. What benefit or advantages do you perceive for the use of a risk assessment based on someone’s [family history of/ genetic predisposition for] diabetes? What is the most important benefit or advantage?

3. What drawbacks or disadvantages do you perceive for such an assessment? What is the most important drawback or disadvantage?

*Elaborate if it does not come up: possible impact of both tests on well-being, family relations, privacy*

**Vignettes**

**Pete** is 55 years of age. A **family history assessment** has shown that he has an increased risk of getting diabetes, because his mother and sister have diabetes.

**Jan** is also 55 years of age. He had a **DNA test** done and the result has shown that he has an increased risk of getting diabetes, because of a genetic predisposition.

1. In what way do you perceive a difference between Pete and Jan concerning their risk of getting diabetes, or do you see no difference?

2. Who of the two gentlemen, do you think, will be more likely to change his behaviour, thus be more physically active and eat healthier? And why?

3. Would you have a diabetes risk test yourself? What test would you prefer (DNA test or family history assessment)? And why?

*Elaborate if it does not come up: possible impact of both tests on people’s freedom to life their live the way they want to, feelings of guilt or blame by creating expectations, discrimination.*
